# Quantifying lymphocyte vacuolization serves as a measure of CLN3 disease severity

**DOI:** 10.1002/jmd2.12128

**Published:** 2020-06-02

**Authors:** Willemijn F. E. Kuper, Marlies Oostendorp, Brigitte T. A. van den Broek, Karin van Veghel, Lourens J. P. Nonkes, Edward E. S. Nieuwenhuis, Sabine A. Fuchs, Tineke Veenendaal, Judith Klumperman, Albert Huisman, Stefan Nierkens, Peter M. van Hasselt

**Affiliations:** ^1^ Department of Metabolic Diseases, Wilhelmina Children's Hospital University Medical Center Utrecht, Utrecht University Utrecht the Netherlands; ^2^ Department of Clinical Chemistry University Medical Center Utrecht Utrecht the Netherlands; ^3^ Sylvia Toth Center for the Multidisciplinary Follow up of Lysosomal Storage Disorders University Medical Center Utrecht, Utrecht University Utrecht the Netherlands; ^4^ Laboratory of Translational Immunology University Medical Center Utrecht, Utrecht University Utrecht the Netherlands; ^5^ Department of Gastroenterology Wilhelmina Children's Hospital, University Medical Center Utrecht, Utrecht University Utrecht the Netherlands; ^6^ Section Cell Biology, Center for Molecular Medicine University Medical Center Utrecht, Utrecht University Utrecht the Netherlands

**Keywords:** CLN3 disease, flow cytometry, ImageStream, lymphocyte vacuolization, lysosomal membrane‐associated protein‐1 (LAMP‐1), neuronal ceroid lipofuscinosis (NCL)

## Abstract

**Background:**

The CLN3 disease spectrum ranges from a childhood‐onset neurodegenerative disorder to a retina‐only disease. Given the lack of metabolic disease severity markers, it may be difficult to provide adequate counseling, particularly when novel genetic variants are identified. In this study, we assessed whether lymphocyte vacuolization, a well‐known yet poorly explored characteristic of CLN3 disease, could serve as a measure of disease severity.

**Methods:**

Peripheral blood obtained from healthy controls and CLN3 disease patients was used to assess lymphocyte vacuolization by (a) calculating the degree of vacuolization using light microscopy and (b) quantifying expression of lysosomal‐associated membrane protein 1 (LAMP‐1), using flow cytometry in lymphocyte subsets as well as a qualitative analysis using electron microscopy and ImageStream analysis.

**Results:**

Quantifying lymphocyte vacuolization allowed to differentiate between CLN3 disease phenotypes (*P* = .0001). On immunofluorescence, classical CLN3 disease lymphocytes exhibited abundant vacuole‐shaped LAMP‐1 expression, suggesting the use of LAMP‐1 as a proxy for lymphocyte vacuolization. Using flow cytometry in lymphocyte subsets, quantifying intracellular LAMP‐1 expression additionally allowed to differentiate between infection and storage and to differentiate between CLN3 phenotypes even more in‐depth revealing that intracellular LAMP‐1 expression was most pronounced in T‐cells of classical‐protracted CLN3 disease while it was most pronounced in B‐cells of “retina‐only” CLN3 disease.

**Conclusion:**

Lymphocyte vacuolization serves as a proxy for CLN3 disease severity. Quantifying vacuolization may help interpretation of novel genetic variants and provide an individualized readout for upcoming therapies.

**Synopsis**

Quantifying lymphocyte vacuolization—either manually or by determining flow cytometry‐derivedlysosomal‐associatedmembrane‐1expression—serves as a measure of CLN3 disease severity.

## 1 INTRODUCTION

CLN3 disease (OMIM #204200) is the most common of the neuronal ceroid lipofuscinoses (NCLs), a genetically heterogeneous group of lysosomal storage disorders (LSDs) unified by the accumulation of autofluorescent storage material in all tissues with the retina and the brain as the predominantly affected tissues.^1^ CLN3 disease is caused by biallelic mutations in *CLN3*, encoding a transmembrane protein involved in lysosomal homeostasis. While the retinal phenotype is relatively uniform throughout the disease spectrum, it is known that the severity of mutations in *CLN3* determines the neurocognitive consequences of the disease.^2^, ^3^, ^4^, ^5^

Abundant lymphocyte vacuolization in a school‐aged child suffering from retinal dystrophy is pathognomonic for (classical) CLN3 disease.^6^, ^7^, ^8^ Differentiation from controls may, however, be difficult, as lymphocyte vacuolization—to a certain, yet unspecified degree—could also be due to a physiological response to a recent infection.^9^ Differentiation from controls may be particularly difficult in nonclassical forms of the disease^10^ that with the increasing use of untargeted genetic analyses are increasingly being identified.^3^, ^11^

We hypothesized that quantifying lymphocyte vacuolization would provide an objective diagnostic marker that simultaneously allows to assess disease severity.

## 2 METHODS

### 2.1 Study population

Peripheral blood samples left over after routine analyses were obtained from patients with genetically confirmed CLN3 disease (Table 1) at diagnosis and during follow‐up and from five patients with other LSDs at one occasion. This latter cohort comprised two patients with NCL subtypes in which lymphocyte vacuolization is known to be absent (one patient with variant juvenile CLN1 disease, one patient with variant late infantile CLN5 disease) and three patients with other LSDs associated with lymphocyte vacuolization (two with sialidosis type I, one with alpha‐mannosidosis).^6^

**TABLE 1:** overview of CLN3 disease patients and samples

No. patients	CLN3 genotype	Phenotype	No. peripheral blood samples
*Classical CLN3 disease* (genotype consisting of two truncating mutations)			
10	1 kb deletion in homozygous form	Childhood onset retinal dystrophy Childhood onset neurodegeneration	29
1	Deletion of exons 9 to 15 in homozygous form	Childhood onset retinal dystrophy Childhood onset neurodegeneration	7
1	c.1054C > T nonsense mutation in homozygous form	Childhood onset retinal dystrophy Childhood onset neurodegeneration	8
2	1 kb deletion and delG561 in exon 6	Childhood onset retinal dystrophy Childhood onset neurodegeneration	2
1	1 kb deletion and c.379delC	Childhood onset retinal dystrophy Childhood onset neurodegeneration	1
*Delayed classical CLN3 disease* (genotype consisting of one truncating mutation and one relatively “mild” missense mutation)			
1	1 kb deletion and c.1000C > T missense mutation	Childhood onset retinal dystrophy Adolescence onset neurodegeneration	5
*Protracted CLN3 disease* (genotype consisting of at least one relatively “mild” missense mutation)			
1	1 kb deletion and c.1A > C missense mutation	Discussed in Reference 18	10
1	c.139 T > C missense mutation and c.1000C > T missense mutation	Childhood onset retinal dystrophy Late adolescence‐adult onset neurodegeneration	2
*CLN3‐associated retinal degeneration* (genotype consisting of two particularly “mild” missense mutations)			
1	c.1213C > T missense mutation in homozygous form	Discussed in Reference 10	3

### 2.2 Controls

Clinically relevant control peripheral blood samples were obtained from six children in whom the diagnosis of CLN3 disease was ruled out: three patients whose retinal dystrophy was found to have a different cause (in two patients biallelic mutations in *ABCA4* were found associated with Stargardt disease;^12^ in the third patient, biallelic mutations in *CEP83* were found associated with a ciliopathy,^13^ and three siblings of CLN3 disease patients who turned out to be heterozygous carriers of the common 1kb deletion in *CLN3*).

Additional blood samples were collected from 22 healthy controls (10 used for peripheral blood smear analysis, 10 for flow cytometry, 1 for ImageStream, and 1 for immunoelectron microscopy).

### 2.3 Assessment of lymphocyte vacuolization

#### 2.3.1 Routine automated hematology analysis

Complete blood count with standard five‐part leucocyte differentiation was performed using three commonly applied hematology analyzers that use optical light scatter (Abbot Cell‐Dyn Sapphire and Sysmex XE‐2100) and impedance technology (Abbot Cell‐Dyn 1800) to differentiate between leucocyte subclasses.^14^, ^15^

### 2.4 Peripheral blood smear analysis

Peripheral blood smears were prepared using an automated slide maker stainer (Abbott Cell‐Dyn SMS) and stained with May‐Grünwald‐Giemsa. It is known that manual assessment is complicated by the heterogeneity between affected and nonaffected cells as only a proportion of lymphocytes is vacuolated in CLN3 disease,^6^, ^7^ and the heterogeneity between affected cells as vacuolated lymphocytes may have different appearances, even within the same (classical) CLN3 disease patient (Supplemental Figure S2A). To account for this, three experienced laboratory technicians independently counted the percentage of vacuolated lymphocytes in a total of 100 lymphocytes and the number of vacuoles per lymphocyte in a maximum of 20 vacuolated lymphocytes.

### 2.5 Flow cytometry staining and flow cytometry

Lysated whole blood (EDTA anticoagulated) was incubated using directly labeled monoclonal antibodies against surface proteins (anti‐CD56 BD Biosciences diluted 1:100; anti‐CD20 BD Biosciences diluted 1:100; anti‐CD3 BD Biosciences diluted 1:50; anti‐CD4 Sony Biotechnology diluted 1:50; anti‐CD8 Biosciences diluted 1:50) and intracellular proteins (anti‐lysosomal‐associatedmembrane‐1 [LAMP‐1] [CD107a] BD Biosciences diluted 1:25) and anti‐perforin eBioscience diluted 1:50). An additional panel was constituted in which anti‐LAMP‐1 was replaced by anti‐LAMP‐2(CD107b BD Biosciences diluted 1:25). Directly following staining, cells were acquired on FACSCanto II and analyzed using FACS Diva Version 6.13 (BD Biosciences) or FlowJo version 7.6.5 software. Within the lymphocyte population, B‐lymphocytes and T‐lymphocytes were differentiated based respectively on CD20 and CD3 fluorescence. T‐lymphocytes were further differentiated into CD4 and CD8 positive T‐cells. Non‐B/T CD56 positive cells were categorized as NK cells. Gating for intracellular lysosomal accumulation was based on the NK cell population used as an internal positive control for both increased LAMP and perforin expression. To determine the degree of lysosomal accumulation, the percentage of affected cells—defined as the subset of cells positive for LAMP expression but negative for perforin expression—was measured for each lymphocyte subset.

### 2.6 ImageStream

Peripheral blood samples (EDTA anticoagulated) collected from one classical CLN3 disease patient and one healthy control were incubated using directly labeled monoclonal antibodies against surface proteins (anti‐CD3 BD Biosciences diluted 1:10; anti‐CD4 Invitrogen diluted 1:50) and intracellular proteins (anti‐LAMP‐1 [CD107a] BD Biosciences diluted 1:25; anti‐perforin eBioscience diluted 1:50). Expression of surface proteins and intracellular proteins in lymphocytes was measured by ImageStream (Amnis, Seattle, Washington). A minimum of 10 000 events were acquired for this analysis. Gating of lymphocytes was performed using the IDEAS software (v6.2). Subsequently, all images of CD4 positive lymphocytes were extracted as 8‐bit Tagged image file format images in a channel separated manner (LAMP‐1, CD4) and imported in a customized script written in Python (Python v2.7) that was used to further process the images. First, an Otsu's thresholding method‐based segmentation approach was used to obtain whole‐cell masks of all lymphocytes. Hereby cell segmentation boundaries were based on the (surface) CD4 signal, as expressed in the CD4 channel. Using these segmentation, masks the mean LAMP‐1 expression for each individual cell was calculated. Additionally, using a “sliding threshold” procedure the distribution of LAMP‐1 expression in all lymphocytes was assessed. Thus, for each grayscale intensity level (0‐255, 8‐bit) the percentage of cell surface that had a LAMP‐1 intensity equal or above this intensity level was calculated. A total of 10 492 CD4 positive lymphocytes were analyzed (healthy control: 2364 cells, classical CLN3 disease: 8128 cells).

### 2.7 Immunoelectron microscopy

Peripheral blood samples (sodium‐heparin anticoagulated) collected from two classical CLN3 disease patients and one healthy control were mixed with equal amounts of PBS containing 2% FBS and centrifuged at RT for 10 minutes at 600 g without brake. A concentrated leukocyte band (buffycoat) was collected and transferred to freshly prepared fixative containing 2% formaldehyde and 0.2% glutaraldehyde in 0.1 M phosphate buffer pH 7.4 for 10 minutes, refreshed to continue fixation for 2 hours. Cells were stored in 1% formaldehyde at 4°C at least overnight, after which a second leucocyte concentration step was performed. Processing of cells for ultrathin cryosectioning and immunolabeling according to the protein A‐gold method was performed as described previously.^16^ In brief, fixed cells were washed with 0.05 M glycin in PBS, resuspended and pelleted in 12% gelatin in PBS at 37°C. The cell pellet was solidified on ice and cut into small blocks. For cryoprotection, blocks were infiltrated overnight with 2.3 M sucrose at 4°C, then mounted on aluminum pins and frozen in liquid nitrogen. A 1:1 mixture of 2.3 M sucrose and 1.8% methylcellulose was used to pick up the ultrathin cryosections (60 nm). Grids were labeled with anti‐LAMP‐1(CD107A BD Biosciences) diluted 1:150 in PBS, 0,1% BSA‐C + 0.5% FSG. Followed by protein A conjugated to 10 nm gold particles was homemade in UMC Utrecht (www.cellbiology-utrecht.nl/second-menu-cmc/products.html), diluted 1:50.

### 2.8 Statistical analysis

Patient characteristics were reported as frequencies and percentages for categorical variables and mean (SD) or median (range) for continuous variables. To analyze the effect of age as a proxy for disease progression on the percentage of vacuolated lymphocytes, and percentage of single positive LAMP‐1 CD4, CD8, and CD20 cells, a linear mixed model was fitted with age and CLN3 phenotype as fixed effect. Based on the Akaike Information Criterion, a random intercept was included per individual to account for individual variation in percentage of positive cells at baseline and a random slope (for age) for dependency across the repeated measurements within the same individual during follow‐up. Final coefficients were estimated using restricted maximum likelihood. The model assumptions including normal distributed residuals, random effects, and homogeneity of variance were confirmed visually.

*P*‐values <.05 were considered significant. The R project for statistical computing version 3.4.1 was used for all mixed model analyses using the packages “nlme” and “ggplot2.”^17^

## 3 RESULTS

### 3.1 CLN3 disease patients

Between 2012 and 2019, we collected 67 peripheral blood samples from 19 patients with genetically confirmed CLN3 disease comprising the complete phenotypic spectrum of the disease^2^, ^3^, ^10^, ^18^ (Table 1).

### 3.2 Quantifying lymphocyte vacuolization

**FIGURE 1:**
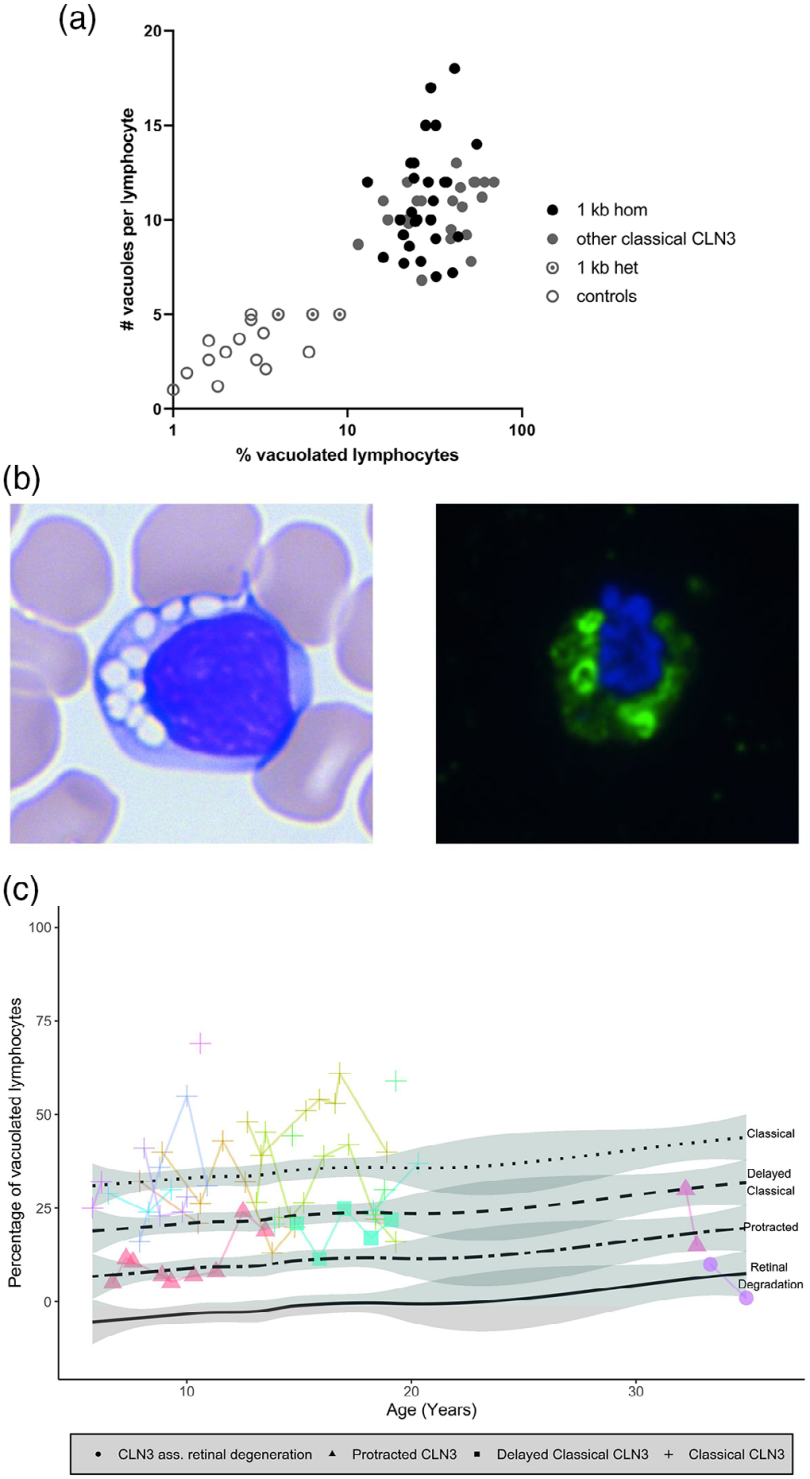
A, Quantifying lymphocyte vacuolization in healthy controls compared to (classical) CLN3 disease. Quantifying the degree of lymphocyte vacuolization—in this study performed along two axes: the percentage of vacuolated lymphocytes (log 10) and the number of vacuoles per affected lymphocyte (linear)—accurately distinguishes (classical) CLN3 disease patients from healthy controls. Among the healthy controls are some individuals who are heterozygous for the common 1 kb deletion in *CLN3* in whom the degree of vacuolization appears slightly higher. B, Microscopical assessment of lymphocyte vacuolization in CLN3 disease. Left: light microscopy image of a vacuolated lymphocyte in classical CLN3 disease. Right: ImmunoFluorescence on 300 nm thick sections of a lymphocytes of a classical CLN3 disease patient (not the same cell as on the left panel) displaying abundant LAMP‐1 expression (in green) appearing at the membranes of the vacuoles surrounding the nucleus (in blue). C, Quantifying lymphocyte vacuolization in different CLN3 disease severity phenotypes. The percentage of vacuolated lymphocytes with a single positive LAMP‐1 expression measured on flow cytometry. Connected lines show repeated measurements per patient. The linear mixed model estimates stable percentages of singe positive vacuolated lymphocytes per CLN3 disease severity type over time (*P* = .26) which is represented by the black lines. LAMP‐1 = lysosomal‐associated membrane protein 1

Routine complete blood count analysis failed to detect vacuolated lymphocytes (Supplemental Figure S1A‐C). Therefore, we next assessed lymphocyte vacuolization semiquantitatively through manual peripheral blood smear analysis. Lymphocyte vacuolization was rare with 1% to 3% of lymphocytes exhibiting some degree of vacuolization in both healthy controls and patients suffering from retinal dystrophy not caused by CLN3 disease, a relevant reference population (Figure 1A). The few (one to five per lymphocyte) vacuoles present were small and difficult to discern (Supplemental Figure S2B). In contrast, vacuolization was abundant in patients with classical CLN3 disease (Figure 1A,B; Supplemental Figure S2B) with 11% to 69% of lymphocytes exhibiting vacuolization (median 30%). The vacuoles in these patients were usually large, with the number per lymphocyte ranging from 6 to 18 (median 10). Of interest, patients with a milder disease severity exhibited fewer and smaller vacuoles (Supplemental Figure S2B). The percentage of lymphocytes exhibiting vacuolization in patients with protracted CLN3 was 5% to 30% (median 8%), with a median of eight vacuoles per lymphocyte (ranged 3‐12). In support of a gene dose effect, mixed model analysis which allows correction for repeated measurements, showed a statistically significant increase in vacuolization with increasing disease severity (*P* = .0001; Figure 1C). The degree of vacuolization was not associated with age, or longer follow up duration, arguing against vacuolization as a proxy for CLN3 disease progression (*P* = .26; Figure 1C).

We next explored if the combination of both discriminative parameters (percentage of vacuolated lymphocytes and number of vacuoles per lymphocyte) could aid further delineation of CLN3 phenotypic severity, even per genotype. As depicted in Figure 1A, this visual analysis allowed to clearly differentiate patients homozygous for the common 1 kb deletion from controls. Interestingly, even within the classical phenotype, patients harboring the severest mutations in *CLN3* (large deletions and frameshift variants) appeared to have the highest percentage of vacuolated lymphocytes (Supplemental Figure S3A). Conversely, each of the patients with a protracted phenotype exhibited less abundant vacuolization to a degree that aligned with the clinical severity of each individual patient, being closer to the classical range in patients with a slightly protracted disease course while close to or even within average control values in the mildest CLN3‐associated retinal degeneration patient (Supplemental Figure S3B).

Vacuoles in CLN3 disease are considered to be of lysosomal origin. We thus hypothesized that increased vacuolization is associated with a larger lysosomal compartment; hence, an increased degree of lysosomal membrane protein expression. Indeed, using immunofluorescence on classical CLN3 disease lymphocytes, abundant LAMP‐1 expression was observed in vacuole‐shaped appearances, mirroring the vacuoles observed using light microscopy (Figure 1B). We next used flow cytometry to quantify LAMP‐1 expression.

### 3.3 Quantifying LAMP‐1 expression as a proxy for lymphocyte vacuolization

**FIGURE 2:**
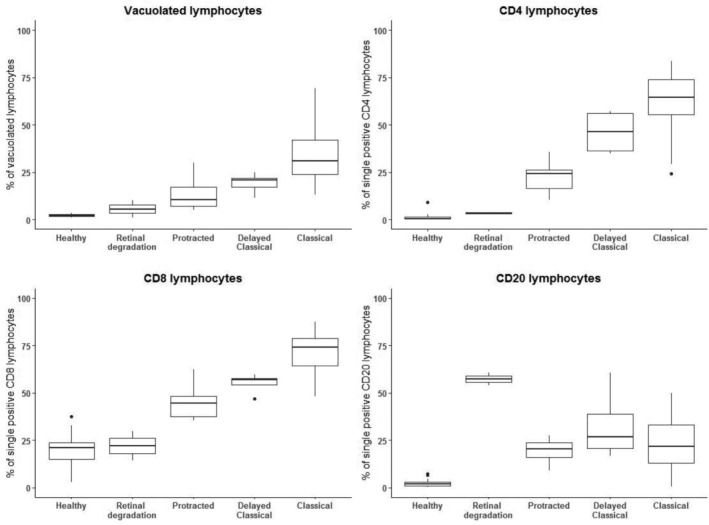
Lymphocyte vacuolization and LAMP‐1 expression correlate with CLN3 disease severity. The percentage of vacuolated lymphocytes (left upper panel), percentage of LAMP‐1 single positive CD4 (right upper panel), CD8 (left lower panel), and CD20 (right lower panel) lymphocytes split per phenotypic subtype shown in bar plots. In the percentage of vacuolated lymphocytes, and single positive CD4 and CD8 lymphocytes a clear correlation with disease severity is seen. In the CD20 subset, a particularly high degree of LAMP‐1 expression is seen in the mildest CLN3 disease phenotype: CLN3‐associated retinal degeneration. LAMP‐1 = lysosomal‐associated membrane protein 1

**FIGURE 3:**
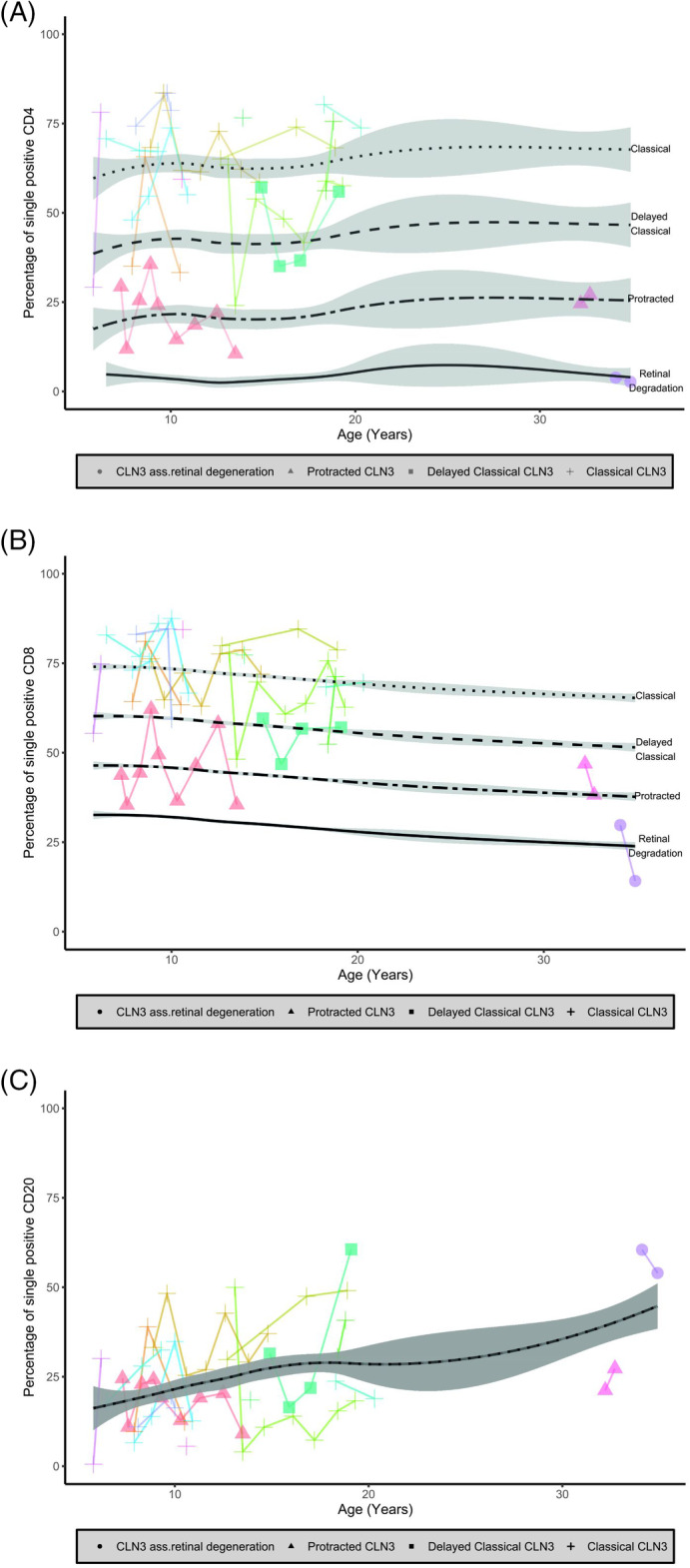
Flow cytometry analyses of LAMP‐1 expression in CLN3 disease in different lymphocyte subsets. The percentage of lymphocytes with a single positive LAMP‐1 expression measured on flow cytometry. A, CD4; B, CD8; and C, CD20. Connected lines show repeated measurements per patient. The linear mixed model estimates stable percentages of singe positive lymphocytes per CLN3 disease severity type over time which is represented by the black lines. LAMP‐1 = lysosomal‐associated membrane protein 1

Intracellular LAMP‐1 expression was generally low in both controls and patients with CLN‐subtypes (CLN1 disease and CLN5 disease) without lymphocyte vacuolization. Any increases of LAMP‐1 expression in these categories were only observed in the CD8 subset coinciding with increased perforin expression together indicating cytotoxic activation. In contrast, CLN3 disease lymphocytes exhibited an increased intracellular expression of both LAMP‐1 (Figure 2) and LAMP‐2 (data not shown), regardless of perforin expression. Mixed model analysis showed that LAMP‐1 expression in both the CD4 and CD8 compartment correlated strongly with CLN3 disease phenotypic severity (*P* < .0001 for both CD4 and CD8) (Figure 3A,B). LAMP‐1 expression did not change over time: expression of LAMP‐1 was stable in all lymphocyte compartments (*P* = .53 for CD4, *P* = .11 for CD8, *P* = .14 for the CD20 without the CLN3‐associated retinal degeneration subtype). Enigmatically, LAMP‐1 expression was markedly—andrepeatedly—elevated in the CD20 compartment of the CLN3‐associated retinal degeneration patient (Figures 2 and 3C). Thus, quantifying LAMP‐1 expression in lymphocytes using flow cytometry outperforms microscopical assessment at the mildest end of the CLN3 disease spectrum.

### 3.4 Explaining the apparent discrepancy between lymphocyte vacuolization and LAMP‐1 expression

**FIGURE 4:**
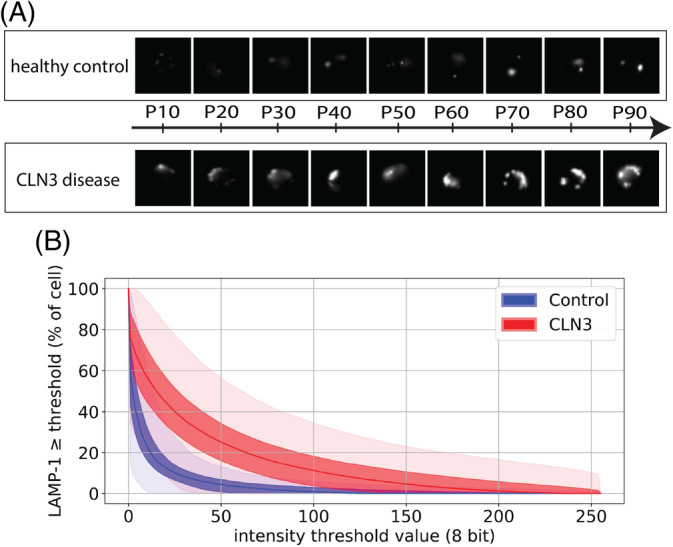
ImageStream analysis of LAMP‐1 expression and distribution CLN3 disease lymphocytes. ImageStream analysis of classical CLN3 disease lymphocytes compared to healthy control lymphocytes confirmed increased expression of LAMP‐1 in CLN3 disease lymphocytes distributed throughout a major area of the cell. A, Examples of LAMP‐1 expression in CD4 positive lymphocytes. The mean LAMP‐1 expression/cell was calculated for all lymphocytes per condition (healthy vs CLN3). Shown are representative examples of mean LAMP‐1 expression/cell at the 10th up to 90th percentile of the distributions. B, Median LAMP‐1 intensity expressed as % of cell above/equal to a specified intensity threshold value (x‐axis), CLN3 disease vs healthy control. Shaded ranges: dark band represents interquartile range (25th‐75th percentile), light band 2.5th to 97.5th percentile range. LAMP‐1 = lysosomal‐associated membrane protein 1

**FIGURE 5:**
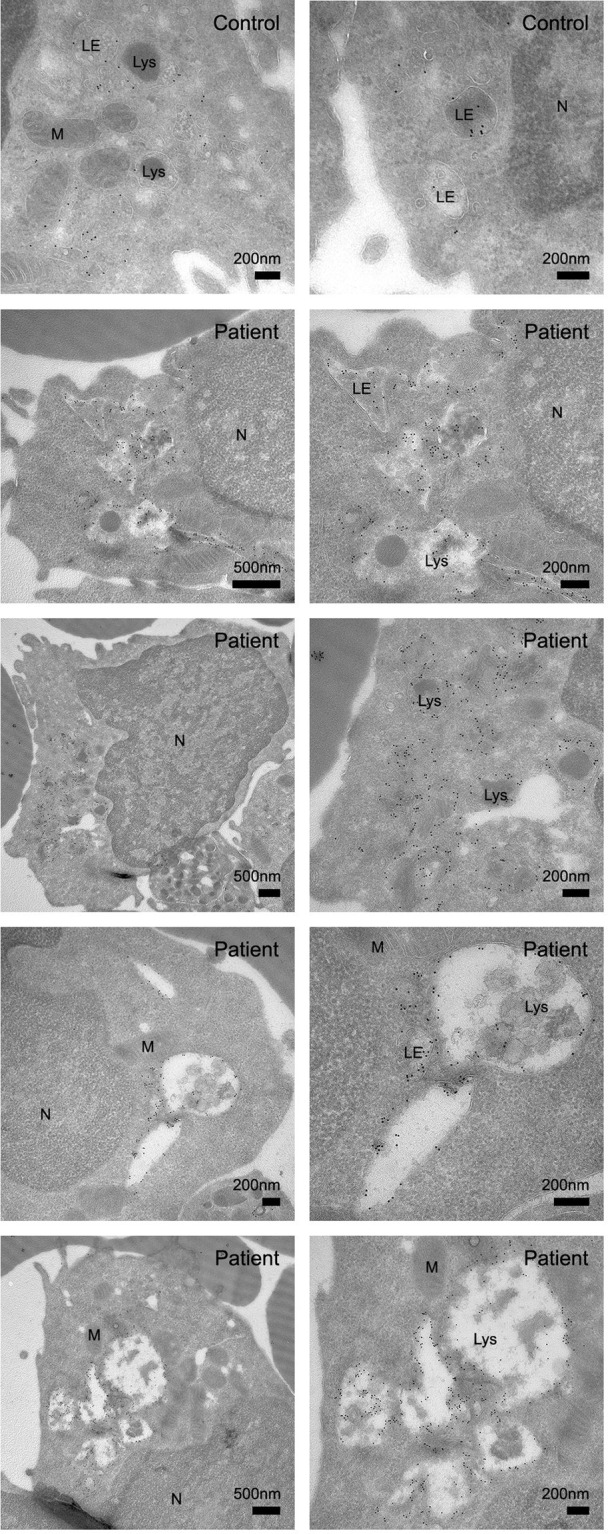
Immunoelectron microcopy imaging of LAMP‐1 expression and distribution in CLN3 disease lymphocytes. Immunoelectron microscopy of ultrathin cryosections labeled with LAMP‐1—10 nm protein A gold particles. Compared to a healthy control, lymphocytes from a classical CLN3 disease patient show an increased LAMP‐1 expression at the membranes of vacuolated lysosomes, but also around nonvacuolated lysosomes and late endosomes. LE = late endosome; Lys = Lysosome; M = mitochondrion; N = nucleus

Contrary to our expectations, we noted that increased LAMP‐1 expression was observed in *all* lymphocytes of patients with CLN3 disease, thus including the many lymphocytes which did not exhibit vacuolization. This observation implies that, rather than a consequence of vacuolization per se, the increased LAMP‐1 expression reflects a more global enlargement of the lysosomal compartment evoked by CLN3 dysfunction. Subsequent ImageStream analysis confirmed that the increased LAMP‐1 expression was found in all lymphocytes and that this increased expression was distributed throughout a major area of the cell (Figure 4). This notion was further supported using immunoelectron microscopy, which showed increased LAMP‐1 expression not only around vacuolated lysosomes but also around nonvacuolated lysosomes and late endosomes (Figure 5), indicative of an overall enlarged lysosomal compartment.

### 3.5 Assessment of LAMP‐1 expression in other vacuolization‐associated disorders

We suspected that the expression of LAMP‐1 in lymphocytes could also help detect other disorders known to exhibit lymphocyte vacuolization.^6^ While in alpha‐mannosidosis, increased expression of LAMP‐1 was most pronounced in the CD4 and CD8 (T‐cell) compartment (Supplemental Figure S4), increased LAMP‐1 expression was especially profound in the CD20 (B‐cell) compartment of patients with sialidosis (Supplemental Figure S5).

Together, these results indicate that LAMP‐1 expression may be used to aid diagnosis of diseases associated with lymphocyte vacuolization and furthermore implies that its distribution within lymphocyte subsets may represent a disease specific “signature.”

## 4 DISCUSSION

In this study, we demonstrate that quantifying lymphocyte vacuolization—either manually or using FACS analysis—both allows to rapidly diagnose vacuolization‐associated metabolic disease and to classify disease severity, particularly CLN3 disease.

Our findings unveil the potential added value of a flow cytometry‐based assay when assessing a metabolic disease. In our study, flow cytometry allowed us to uncover that LAMP‐1 expression was most pronounced in CD4 and CD8 positive cells (T‐cells) in severe end CLN3 disease while at the mildest end of the disease spectrum, in CLN3‐associated retinal degeneration, LAMP‐1 expression was almost absent in T‐cells while pronounced in CD20 positive cells (B‐cells). This predominant B‐cell involvement was also observed in the two sialidosis type I patients included in this study. Sialidosis type I, a different vacuolization‐associated LSD, is characterized by progressive vision loss due to a (macular cherry red spot type of) retinal dystrophy in addition to gait impairment due to progressive myoclonus and ataxia resembling a spastic paraparesis.^19^ Interestingly, while spastic paraparesis is not a symptom of (classical‐protracted) CLN3 disease, it is present in the CLN3‐associated retinal degeneration patient included in this study.^10^ It is tempting to speculate that this clinical overlap reflects a shared pathophysiological mechanism. Future research is needed to elucidate this. Currently, we have included the LAMP‐1 expression assay in the diagnostic process in our hospital. And we foresee that this assay as well as other flow cytometry‐based assays will play a larger role in the diagnostic process. Particularly, the ability of FACS analysis to create relevant subpopulations, with—potentially—different cellular characteristics is likely to result in assays which allow disease monitoring and potentially also aid in understanding the pathophysiology of metabolic diseases.

Contrary to a previous report, we did not find evidence that lymphocyte vacuolization in CLN3 disease increases in parallel with disease progression.^20^ In our study, any increase with age disappeared when patients were grouped according to the severity of the underlying genotype. Likely, the mild increase observed in the Kimura study—involving a relatively small cohort with a short follow‐upperiod—was either due to interpatient differences in the degree of storage material accumulation related to genotypic severity not yet known at that time^20^, ^21^ or due to intrapatient differences in the degree of storage material accumulation. This variation in storage material levels within a patient may be partly due to interobserver differences in quantifying lymphocyte vacuolization, but, since some degree of variation was also present in our study in the automated measurement of LAMP‐1 expression, it may also be the result of an active balance between build‐up and removal of storage material dependent on residual CLN3 protein activity.

Biomarker identification in humans has some limitations, particularly with rare disorders. Limitations include small patient sample sizes and difficulties in obtaining tissue of interest, aggravated by the strict ethical requirements for using body material from children and/or incapacitated individuals. One approach to overcome this could be to first identify biomarkers in small animal models, such as mice. Indeed, one biomarker identification study in mice reported a (minor) increase in lymphocyte vacuolization with age, in addition to several other potential hematological biomarkers for CLN3 disease.^22^ However, since hematological parameters show major variabilities between and even within different mouse inbred strains^23^ this has—at least for CLN3 disease—not yet led to the identification of clinically relevant biomarkers. Although we acknowledge the contribution of small animal models for fundamental pathophysiology research, we would currently advise the use of human material whenever available—for instance, material leftover from routine analyses as we did in this study—to identify biomarkers relevant for the patient. Subsequently, animal models that more closely resemble the human patient, may be used to apply the human‐relevant biomarkers, thereby providing a crucial link between fundamental research and clinical trials.^24^ This might greatly accelerate therapy development for CLN3 disease as well as other metabolic disorders that are currently without treatment.

In conclusion, quantifying lymphocyte vacuolization allows to diagnose CLN3 disease and to classify disease severity. This may help interpretation of novel genetic variants and may provide an individualized and quantitative readout for upcoming therapies.^25^

## CONFLICT OF INTEREST

The authors declare no potential conflicts of interest.

## AUTHOR CONTRIBUTIONS

Willemijn F. E. Kuper: study design, data acquisition, interpretation of data, statistical analysis, drafting of manuscript. Marlies Oostendorp: data acquisition, interpretation of data, critical revision of the manuscript for intellectual content. Brigitte T. A. van den Broek: data acquisition, interpretation of data, statistical analysis, critical revision of the manuscript for intellectual content. Karin van Veghel: data acquisition, interpretation of data, critical revision of the manuscript for intellectual content. Lourens J. P. Nonkes: interpretation of data, statistical analysis, critical revision of the manuscript for intellectual content. Edward E. S. Nieuwenhuis: interpretation of data, critical revision of the manuscript for intellectual content. Sabine A. Fuchs: interpretation of data, critical revision of the manuscript for intellectual content. Tineke Veenendaal: data acquisition, interpretation of data, critical revision of the manuscript for intellectual content. Judith Klumperman: interpretation of data, critical revision of the manuscript for intellectual content. Albert Huisman: study concept and design, data acquisition, interpretation of data, critical revision of the manuscript for intellectual content. Stefan Nierkens: study concept and design, interpretation of data, critical revision of the manuscript for intellectual content. Peter M. van Hasselt (corresponding author): study concept and design, interpretation of data, critical revision of manuscript for intellectual content.

## ETHICAL APPROVAL STATEMENT

This article does not contain any studies with human or animal subjects performed by the any of the authors. A waiver for ethical review was granted by the Medical Ethical Research Committee of the University Medical Center Utrecht (18‐024).

## Supporting information


**Appendix** **S1:** Supporting information

## Data Availability

The data that support the findings of this study are available from the corresponding author upon reasonable request.
