# Supplementary material for: Quantifying lymphocyte vacuolization serves as a measure of CLN3 disease severity
Source: JIMD Rep. 2020 Jun 2;54(1):87–97. doi: 10.1002/jmd2.12128 (PMC7358670; doi:10.1002/jmd2.12128)

**Quantifying lymphocyte vacuolization serves as a measure of CLN3 disease severity**

Willemijn F.E. Kuper MD; Marlies Oostendorp PhD; Brigitte T.A. van den Broek MD; Karin van Veghel MSc; Lourens J.P. Nonkes PhD; Edward. E.S. Nieuwenhuis MD PhD; Tineke Veenendaal; Judith Klumperman PhD; Albert Huisman PhD; Stefan Nierkens PhD; Peter M. van Hasselt MD PhD

**Supplementary material**

**Supplemental Figure 1A :Automated hematological analyzers do not detect abundant lymphocyte vacuolation in classical CLN3 disease**


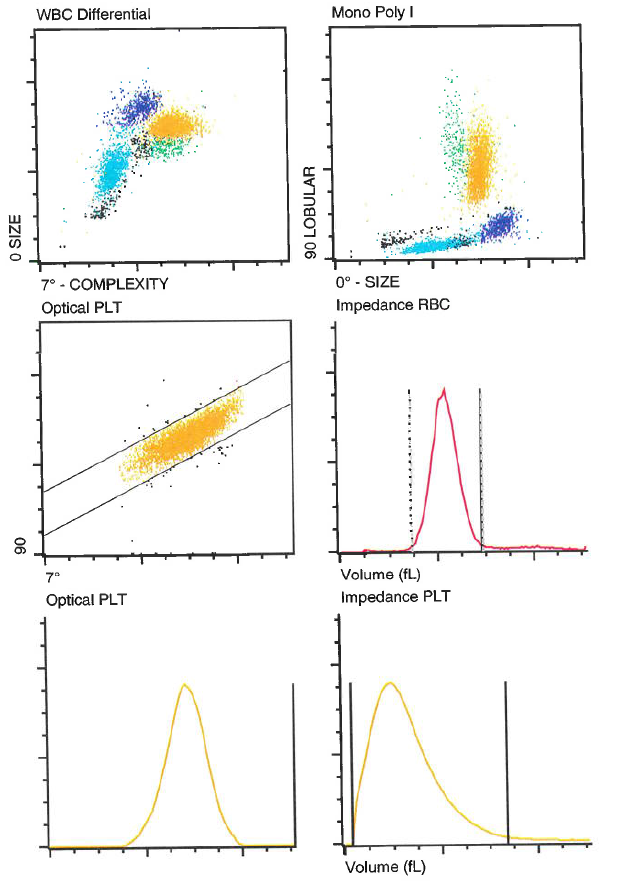


**Supplemental Figure 1B: Automated hematological analyzers do not detect abundant lymphocyte vacuolation in classical CLN3 disease**


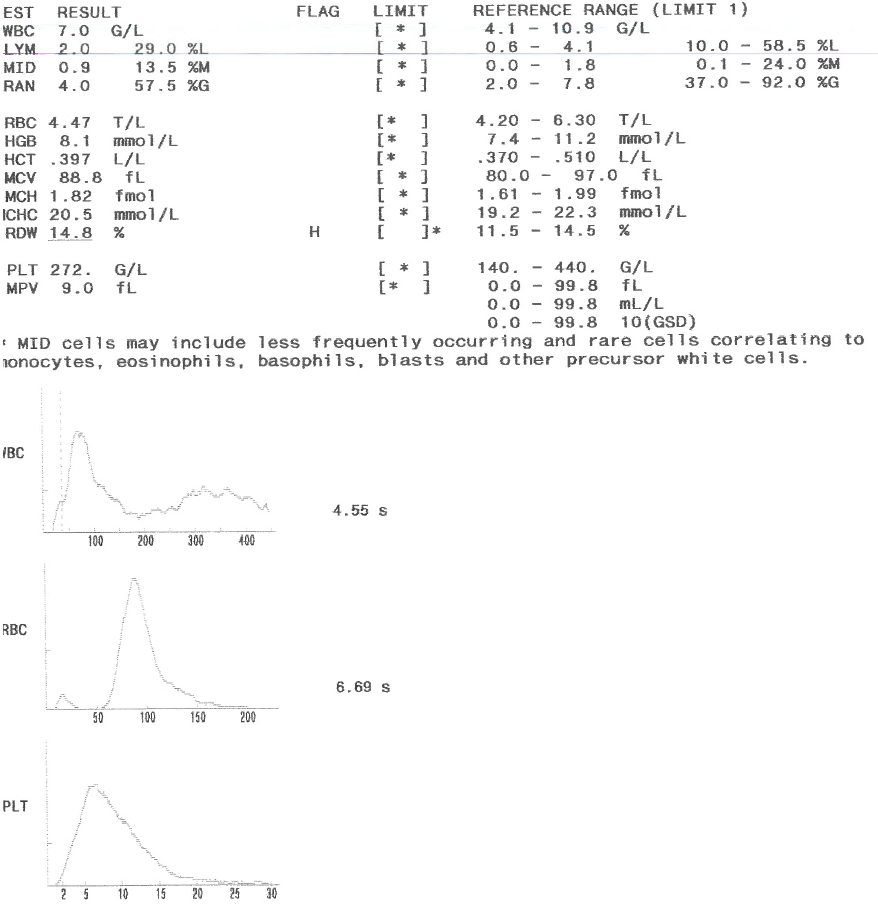


**Supplemental Figure 1C: Automated hematological analyzers do not detect abundant lymphocyte vacuolation in classical CLN3 disease**


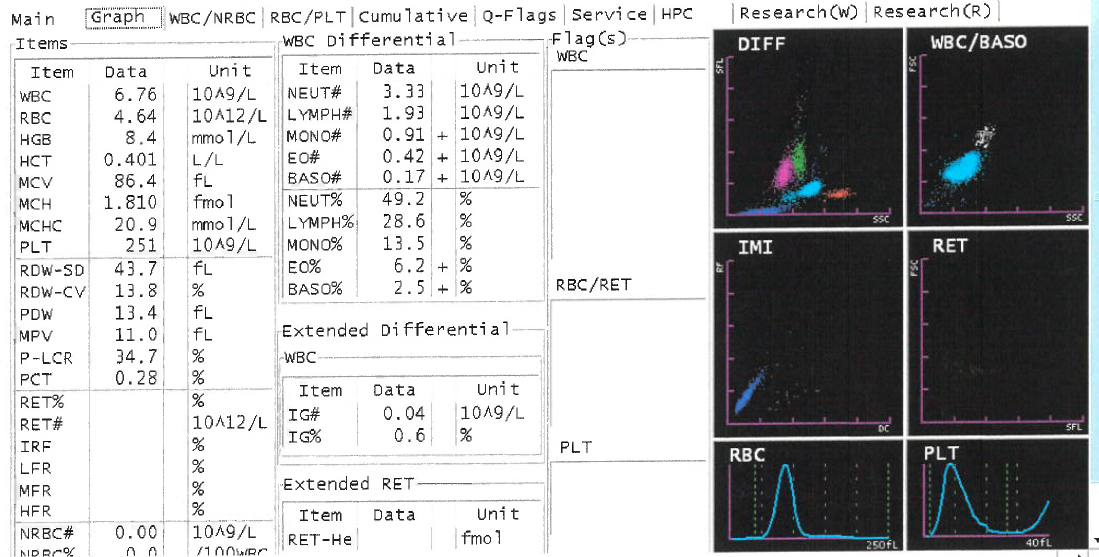
**Supplemental Figure 2: Lymphocyte vacuolization in CLN3 disease**

**A: different manifestations of lymphocyte vacuolization in classical CLN3 disease**

**
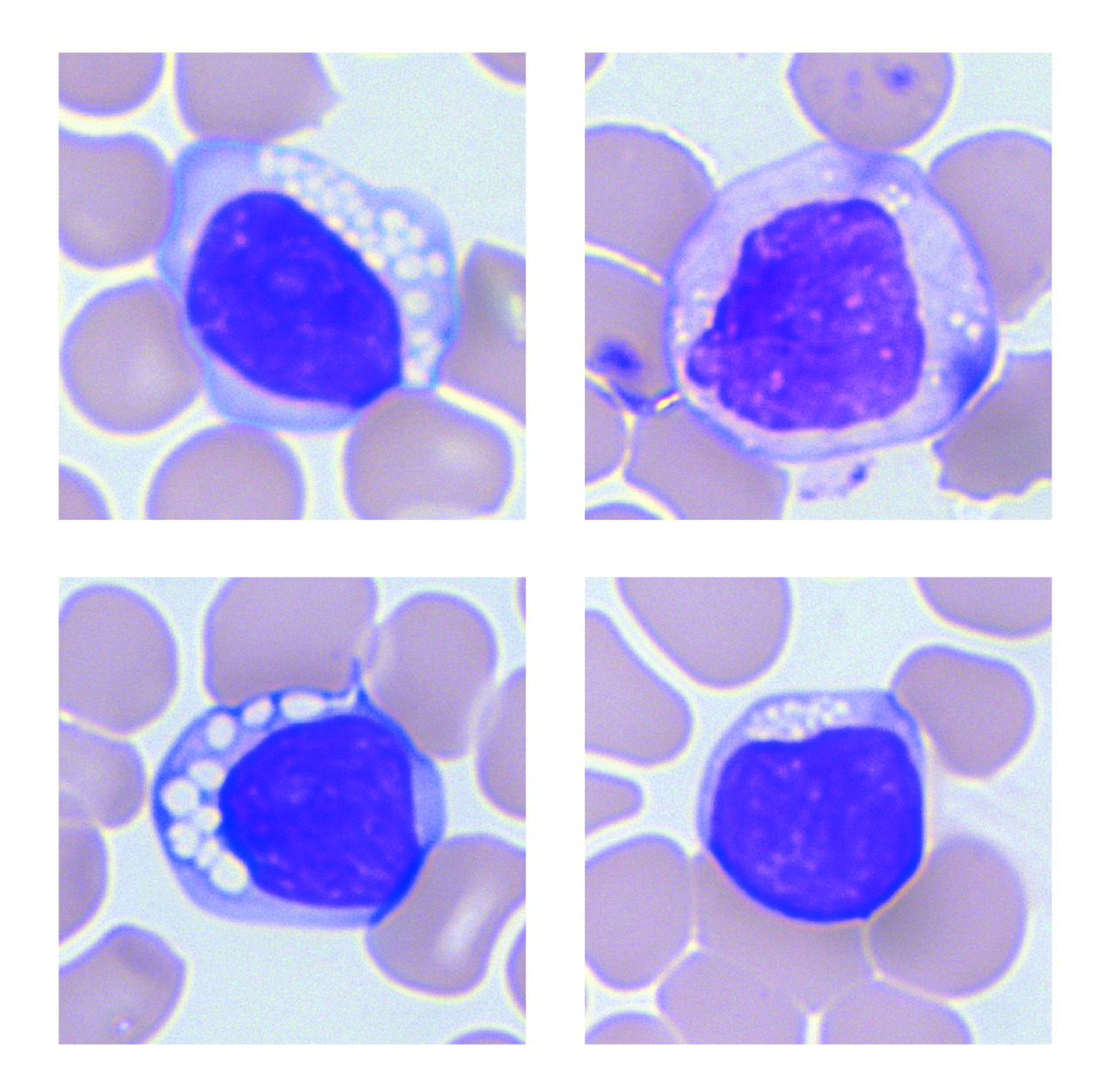
**

Light microcopy image of lymphocyte vacuolization in a classical CLN3 disease patient showing abundant, large vacuoles in some lymphocytes; compared to scattered/ smaller vacuoles in other lymphocytes that are more difficult to detect.

**B: different manifestations of lymphocyte vacuolization among the CLN3 disease severity spectrum**

**
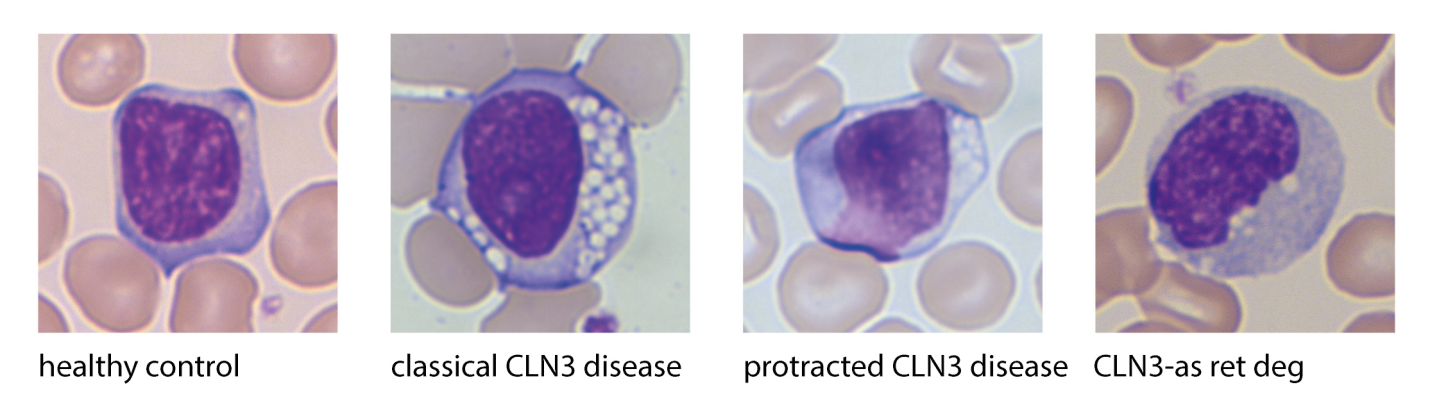
**

Light microcopy image of lymphocyte vacuolization in a healthy control (A) compared to classical CLN3 disease (B), protracted CLN3 disease (C), and CLN3 associated retinal degeneration (D). In CLN3 disease lymphocytes, the degree of vacuolization including the size of the vacuoles appeared to be related to disease severity.

Abbreviations: CLN3-as ret deg = CLN3-associated retinal degeneration

**Supplemental Figure 3: Explorative analysis of the degree of lymphocyte vacuolization in the different CLN3 genotype-phenotype correlations**

**A: classical CLN3 disease**

The number of vacuoles per lymphocyte (linear scale) as compared to the percentage of vacuolated lymphocytes (log10 scale) in the different genotypes associated with classical CLN3 disease. The more severe mutations (i.e. the large exon 9-15 deletion and the frameshift (fs) mutations) appear to be associated with a higher degree of vacuolization.

**B: protracted CLN3 disease and CLN3-associated retinal degeneration**

The number of vacuoles per lymphocyte (linear scale) as compared to the percentage of vacuolated lymphocytes (log10 scale) in two different genotypes associated with protracted CLN3 disease (1 kb deletion / c1.A>C missense mutation and c.1000C>T missense mutation / c.139T>C missense mutation) and one genotype associated with CLN3-associated retinal degeneration (c.1213C>T in homozygous form).

**Supplemental Figure 4: LAMP-1 expression in alfa mannosidosis compared to classical CLN3 disease**

**A: classical CLN3 disease**


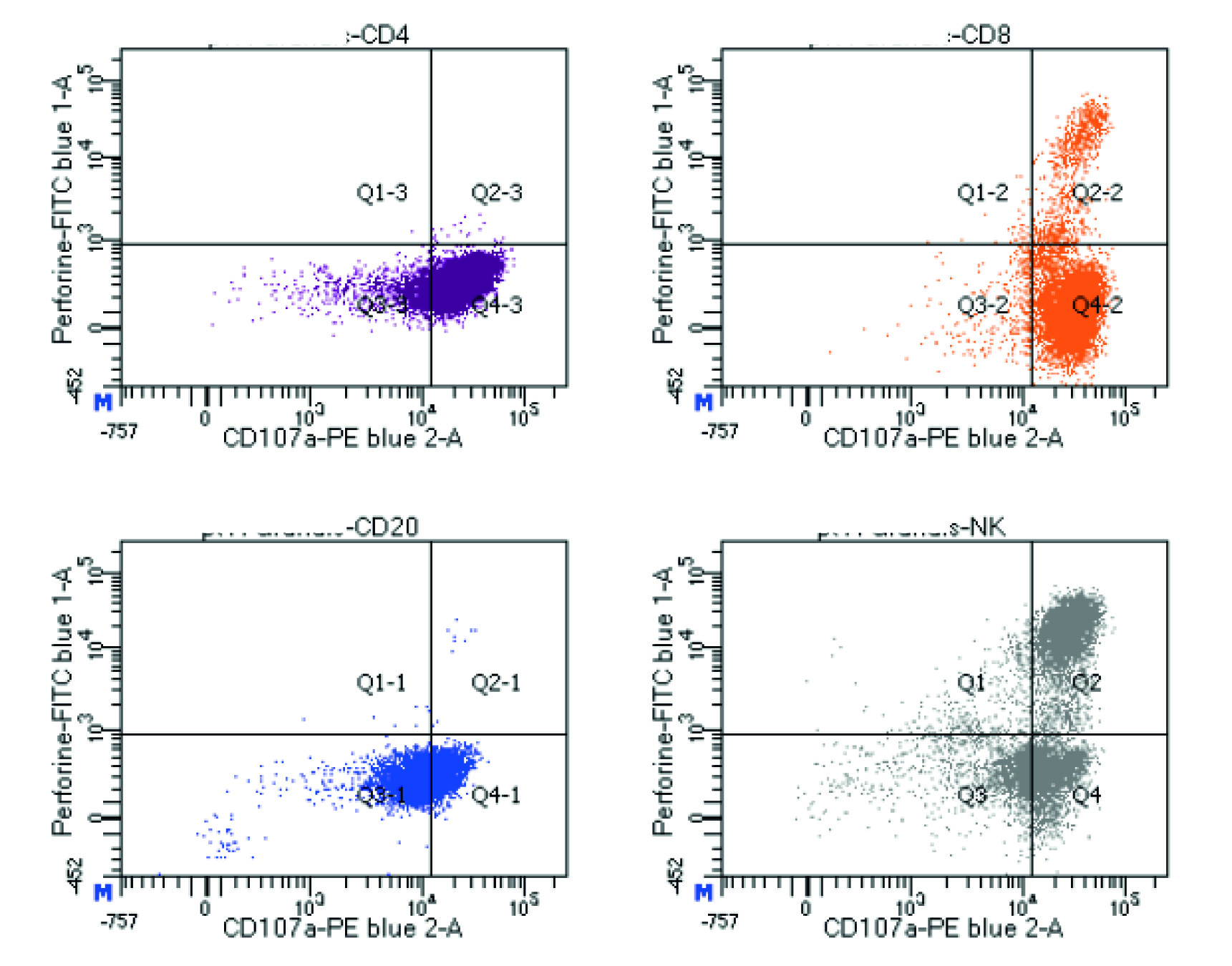


**B: alfa mannosidosis**


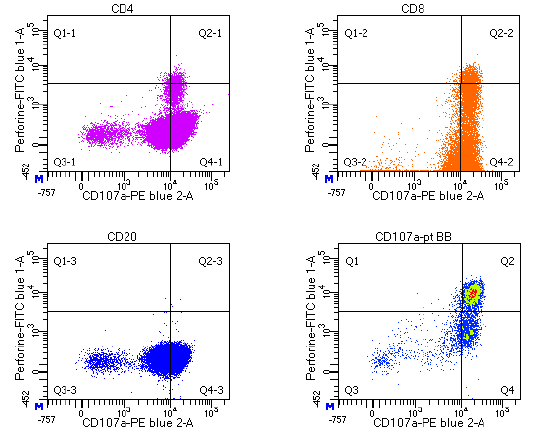


**Supplemental Figure 5: LAMP-1 expression in sialidosis type I compared to CLN3-associated retinal degeneration**

**A: CLN3-associated retinal degeneration**


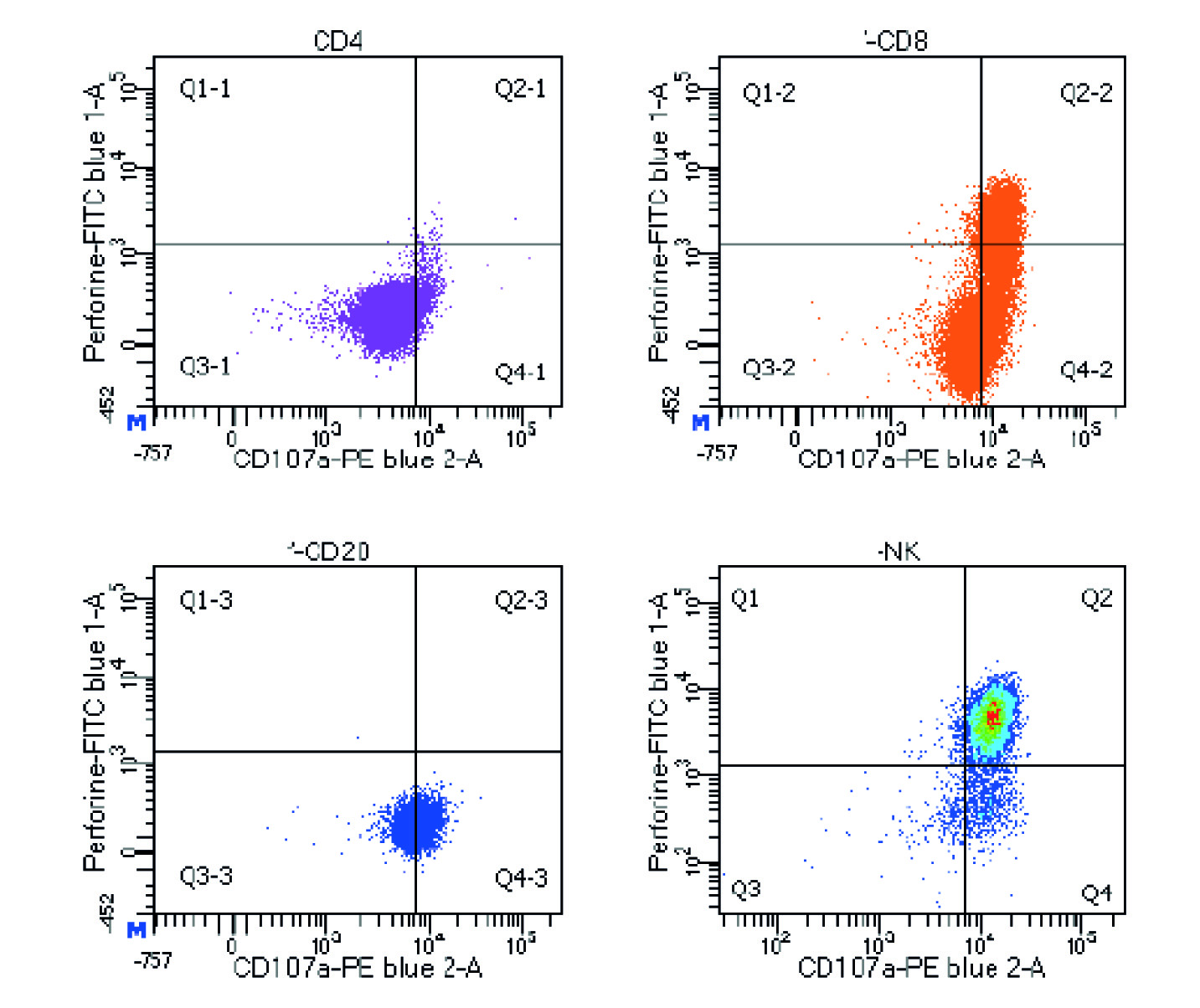


**B: sialidosis type I**


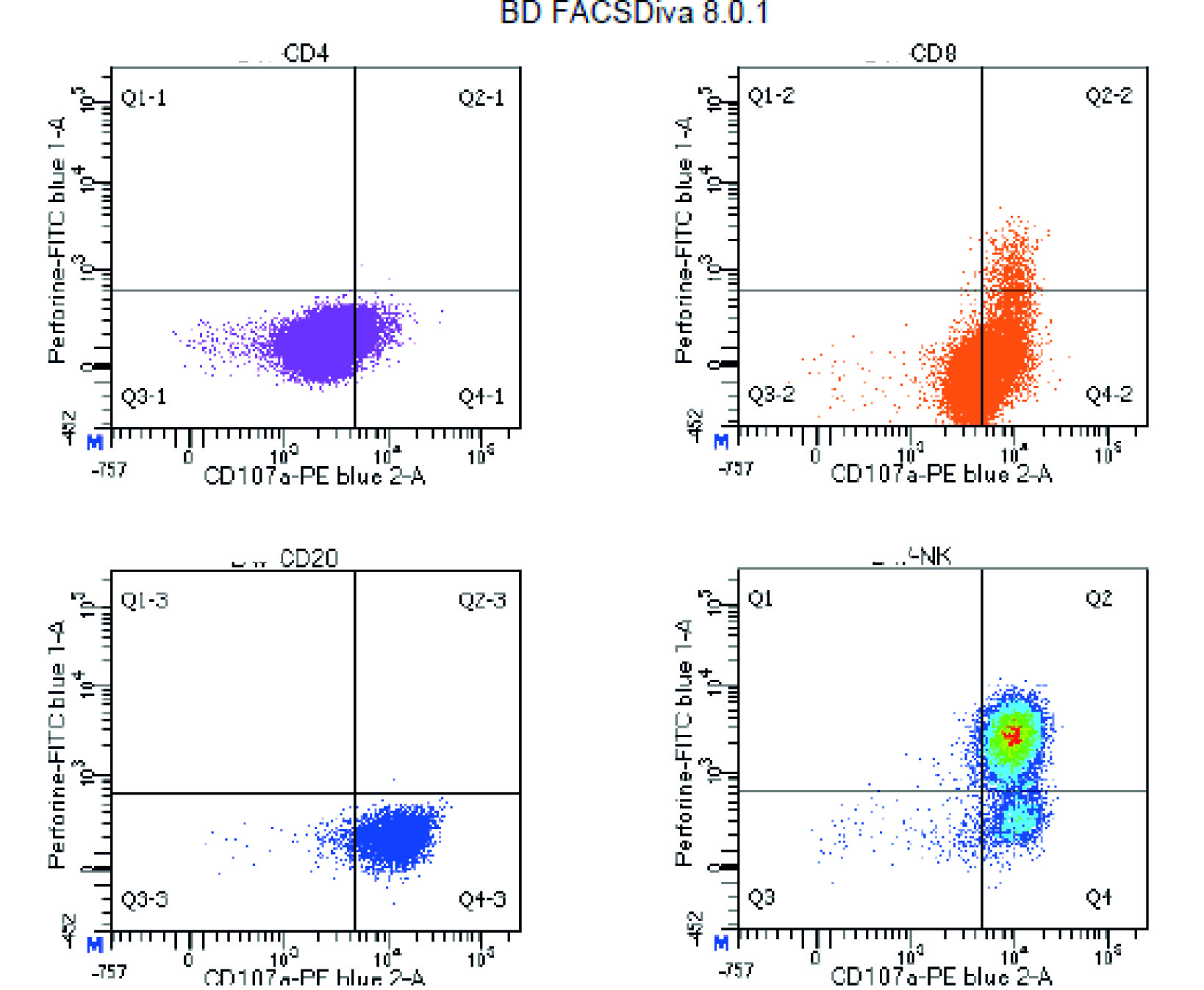

Supplement: Supplementary file 1 — Appendix S1: Supporting information [file JMD2-54-87-s001.docx]
